# Modelling the vertical distribution of canopy fuel load using national forest inventory and low-density airbone laser scanning data

**DOI:** 10.1371/journal.pone.0176114

**Published:** 2017-04-27

**Authors:** Eduardo González-Ferreiro, Stéfano Arellano-Pérez, Fernando Castedo-Dorado, Andrea Hevia, José Antonio Vega, Daniel Vega-Nieva, Juan Gabriel Álvarez-González, Ana Daría Ruiz-González

**Affiliations:** 1 Unidad de Gestión Forestal Sostenible, Departamento de Ingeniería Agroforestal, Universidad de Santiago de Compostela, Lugo, Spain; 2 Departamento de Ingeniería y Ciencias Agrarias, Universidad de León, Campus de Ponferrada, Ponferrada, Spain; 3 Sustainable Forest Management Area, Forest and Wood Technology Research Centre (CETEMAS), Pumarabule, Carbayín, Siero—Asturias, Spain; 4 Centro de Investigación Forestal de Lourizán, Pontevedra, Spain; 5 Instituto de Silvicultura e Industria de la madera, Universidad Juárez del Estado de Durango, Ciudad Universitaria, Durango, Durango, México; Kerala Forest Research Institute, INDIA

## Abstract

The fuel complex variables canopy bulk density and canopy base height are often used to predict crown fire initiation and spread. Direct measurement of these variables is impractical, and they are usually estimated indirectly by modelling. Recent advances in predicting crown fire behaviour require accurate estimates of the complete vertical distribution of canopy fuels. The objectives of the present study were to model the vertical profile of available canopy fuel in pine stands by using data from the Spanish national forest inventory plus low-density airborne laser scanning (ALS) metrics. In a first step, the vertical distribution of the canopy fuel load was modelled using the Weibull probability density function. In a second step, two different systems of models were fitted to estimate the canopy variables defining the vertical distributions; the first system related these variables to stand variables obtained in a field inventory, and the second system related the canopy variables to airborne laser scanning metrics. The models of each system were fitted simultaneously to compensate the effects of the inherent cross-model correlation between the canopy variables. Heteroscedasticity was also analyzed, but no correction in the fitting process was necessary. The estimated canopy fuel load profiles from field variables explained 84% and 86% of the variation in canopy fuel load for maritime pine and radiata pine respectively; whereas the estimated canopy fuel load profiles from ALS metrics explained 52% and 49% of the variation for the same species. The proposed models can be used to assess the effectiveness of different forest management alternatives for reducing crown fire hazard.

## Introduction

Accurate knowledge of fuel characteristics has been shown to be critical in forest fire management, as fuel constitutes a primary component of fire risk [1,2]. Moreover, accurate fuel mapping is required for using fire behaviour models and simulation systems, which are essential for establishing fuel treatment priorities and evaluating the effectiveness of fuel management actions [3].

One of the current main objectives of fuel management in conifer forests is the mitigation of crown fire hazard. Crown fires are usually intense and spread rapidly, which makes them difficult and dangerous to suppress. They can cause severe economic damage and ecological disruption [4].

Assessing the risk of crown fire initiation and spread is a key element in gauging fire behaviour. Both crown fire initiation and spread are widely recognised to be determined by structural characteristics of the canopy fuel complex such as available canopy fuel load (*CFL*), canopy bulk density (*CBD*) and canopy base height (*CBH*) [4]. *CFL* is defined as the mass of available canopy fuel per unit ground area; *CBD* indicates the amount of available fuel per volume unit in the aerial layer, and *CBH* is the lowest height above ground level at which there is sufficient available canopy fuel to propagate fire vertically through the canopy [2,5]. Fire behavior simulation systems that assess crown fire potential thus require accurate estimates of these variables. For modelling purposes, needles and fine twigs are commonly considered available fuel because these tree biomass components are usually consumed within the flaming front of a crown fire [6].

Two main approaches can be used to estimate *CBD* and *CBH* [2,7,8]. The simplest approach is based on the assumption that available canopy fuel is homogeneously distributed throughout the aerial layer of the stand. *CFL* is thus estimated by dividing the total available biomass in the canopy by the stand surface area; *CBD* is estimated by dividing *CFL* by the canopy depth, and *CBH* is estimated as the average crown base height of all trees in the stand. This approach is consistent with the criteria for crown fire initiation and spread proposed by Van Wagner [9] and is integrated in most empirical crown fire models. However, important advances are expected to be made in modelling crown fire behaviour in the future. Therefore, estimation of the vertical distribution of canopy fuels should focus on a more realistic and complex approach based on evidence that the fuel distribution in each tree crown affects the general fuel distribution in the stand [7]. Such a realistic approach assumes that the crown of each tree in the stand has a particular shape that defines the available fuel distribution along the crown. The distribution of the available fuel in the stand is predicted by taking into account the shape of each tree and the height at which the crown starts and ends. *CBD* and *CBH* are subsequently estimated from the predicted vertical distribution according to certain criteria. *CBD* is usually estimated as the maximum bulk density in a vertical profile that shows the distribution of this variable from the ground to the top of the tallest tree in the stand, and *CBH* as the height at which a certain bulk density value is reached in that profile [2]. Different bulk density values were arbitrarily proposed for estimating *CBH*: 0.037 kg m^-3^ [5], 0.012 kg m^-3^ [10] and 0.067 kg m^-3^ [11]. This option enables us to take into account information provided by Airborne Laser Scanning (ALS) data, which can be used to predict the forest structure in three dimensions.

In Spain, the only information currently available regarding canopy fuel complex characteristics at national and regional scales is that provided by the National Forest Inventory (NFI). The NFI is based on a systematic sampling design (1x1 km sample grid) with permanent plots and previous forest stratification by photo-interpretation. However, this type of inventory does not yield full spatial coverage and often takes several years to complete [12]. For example, in the region of Galicia (north-west Spain), the field work involved in the fourth NFI (NFI-4) lasted about 11 months, and data processing took approximately 3 years [13]. Moreover, the NFI is costly and the information provided could become quickly outdated, especially for fast growing species.

ALS has proven to be a useful source of auxiliary data for describing the canopy fuel stratum because it can provide information that can be used to predict the three-dimensional structure of vegetation and other forest features at different scales [14–16]. Research aimed at characterizing forest resources by use of commercial ALS sensors operating at moderate to low spatially dense sampling (up to 0.5 pulses m^-2^) has focused on the stand-level approach, which often establishes empirical relationships between metrics estimated from ALS data and field-measured stand variables. Specific stand-level studies that describe the canopy fuel stratum are scarce and often restricted to a few tree species and small sites [17–23]. Some ALS studies have also attempted to predict diameter probability density function parameters; for example, Gobakken and Næsset [24,25] used the Weibull distribution to predict diameter distributions from laser scanner data; Breidenbach et al. [26] applied a generalized linear model (GLM) to estimate the shape and scale parameters of the Weibull distribution by using ALS metrics as predictors; and Thomas et al. [27] applied ALS-based Weibull parameter prediction in different types of forests including coniferous, hardwoods and mixed woods.

Spain now has a wide coverage of coarse resolution (0.5 first returns m^-2^), small-footprint ALS data, thanks to the *Plan Nacional de Ortofotografía Aérea* 2008–2015 (PNOA 2008–2015), which has carried out 5 annual flight surveys covering an area of 406,550 km^2^ (slightly more than 80% of the surface of Spain). The main aim is to produce fine resolution Digital Elevation Models (DEM) and to control the quality of PNOA conventional photogrammetric products. Although PNOA ALS data were not originally conceived for the purpose of characterizing forest vegetation, it is expected that wall-to-wall, georeferenced point clouds could provide accurate estimates of the available canopy fuel profile.

In this study, we aimed to model the vertical distribution of the available canopy fuel load in *Pinus pinaster* Ait. and *Pinus radiata* D. Don stands in Galicia (north-west Spain), by combining extensive (NFI) field and countrywide ALS data sets. Although the study focuses on two species, the method could be applied to other species when new data acquisitions become available. Two different set of models were used to predict the vertical distributions of available canopy fuel loads and were fitted using different sets of predictors: the first based on stand variables calculated from field measurements in the NFI plots and the second based on ALS metrics.

## Material and methods

### Study area

**Fig 1:**
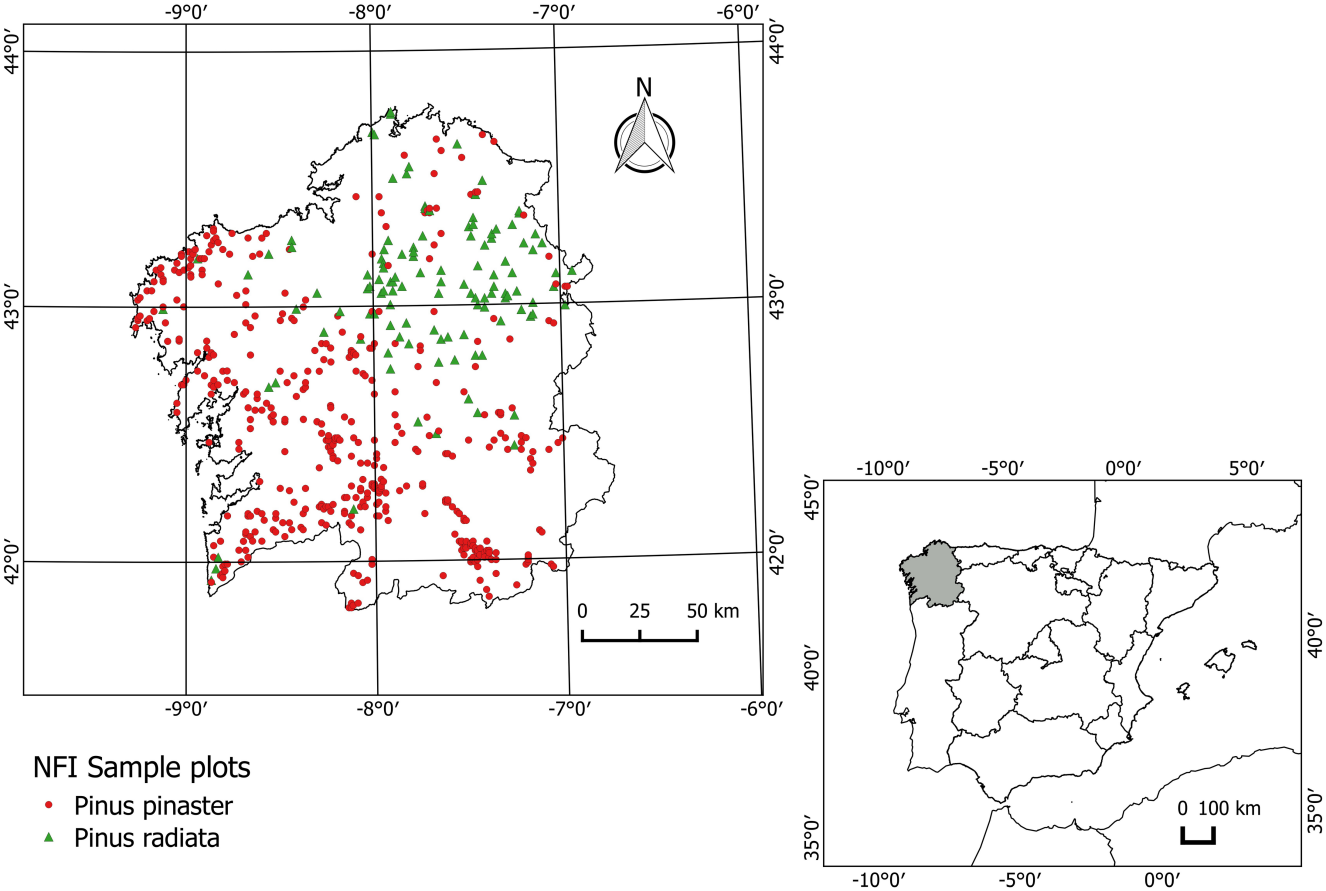
Location of the study area and spatial distribution of the NFI-4 plots where *P*. *pinaster* and *P*. *radiata* were dominant.

The region of Galicia (north-west Spain) is characterized by rugged orography and an oceanic climate with mild temperatures, low thermic oscillation between winter and summer and frequent rainfall. Galicia is one of the most important regions in Spain in terms of forestry production. Around 30% of the 1.4 million hectares of tree-covered land comprises pure and even-aged pine stands, mainly maritime pine (*Pinus pinaster*) (271,281 ha) and radiata pine (*Pinus radiata*) (96,177 ha), and more than 68 million cubic metres of standing timber [13] provide 51% and 27% of respectively the conifer and total annual harvest volume in Spain [28]. Fig 1 shows the location of Galicia in Spain and Europe, and the spatial distribution of pure *P*. *pinaster* and *P*. *radiata* NFI plots.

Pine stands are among the most flammable types of vegetation, particularly when they are not thinned or pruned and carry large surface fuel loads [29,30]. This, together with the low moisture contents reached in the litter in dry summer periods [31], has led to wildfires (especially crown fires) becoming of ecological, economic and social concern in Galicia.

### Field data

**Fig 2:**
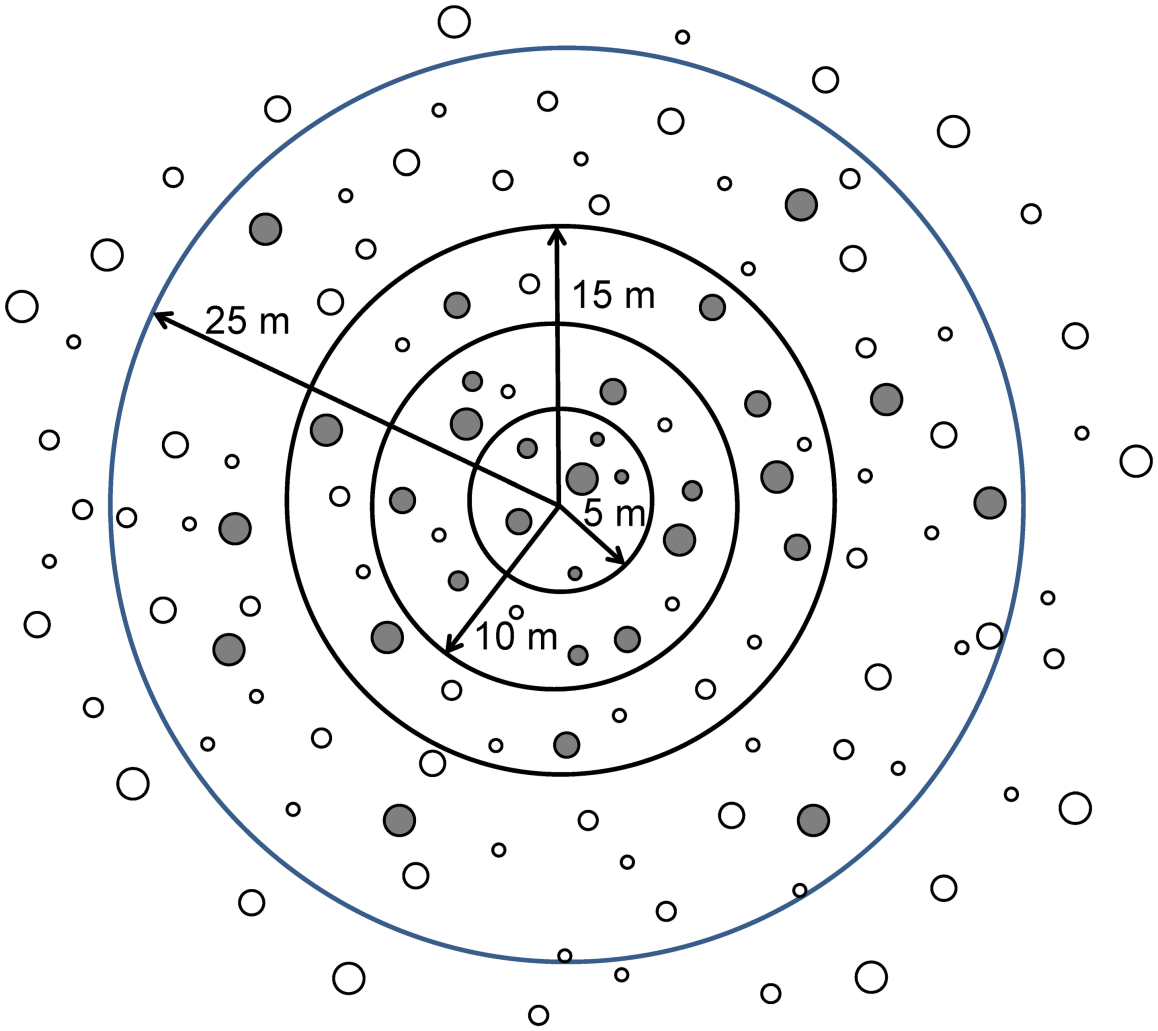
A typical sample plot used in the fourth National Forest Inventory (NFI-4). Grey circles represent trees selected on the basis of tree diameter and the distance to the plot centre.

The field data used for this study were obtained from the NFI-4 carried out in Galicia [13] (data available at http://www.magrama.gob.es/es/biodiversidad/servicios/banco-datos-naturaleza/; last accessed on 7-8-2016). The NFI project maintains a network of sample plots throughout Spain, which are aimed at providing continuously updated information on the status of nationwide forest resources, including timber volumes and species composition [32]. Sample plots are established at the intersections of a 1-km x 1-km UTM grid and consist of four circular concentric subplots of radii 5, 10, 15 and 25 m (Fig 2). Diameter at breast height (*d*) and total tree height (*h*) are measured in trees selected on the basis of their diameter and distance to the plot centre (*d* ≥ 42.5 cm for the 25-m radius; *d* ≥ 22.5 cm for the 15-m radius; *d* ≥ 12.5 cm for the 10-m radius and *d* ≥ 7.5 cm for the 5-m radius). The number of trees of diameter 2.5–7.5 cm (saplings) is also recorded in the 5-m radius subplot. Diameter at breast height is measured to the nearest 0.1 cm with graduated trees calipers in two perpendicular directions. Total tree height is measured to the nearest 0.1 m with a hypsometer. The following stand variables were calculated from the tree variable measurements, by using tree expansion factors: number of stems per hectare (*N*), quadratic mean diameter (*dg*), stand basal area (*G*), mean stand height ($\bar{h}$) and dominant height (*H*, defined as the mean height of the 100 thickest trees per hectare). These variables were used as predictors for statistical analyses. The tree expansion factor adjusts to a per hectare basis for the i*th* tree; it expresses the number of trees per hectare that each selected tree represents in the inventory, according to the subplot radius.

A total of 8515 sample plots were measured in the NFI-4 in Galicia, which corresponds to approximately one sample location per 166 ha of afforested land.

All plots in which *P*. *pinaster* or *P*. *radiata* were dominant (more than 90% of trees and more than 90% of total stand basal area) were selected. In addition, plots in which dead trees accounted for more than 10% of basal area were rejected.

### ALS data

ALS data were acquired under the direction of the Spanish *Ministerio de Fomento* (*Dirección General del Instituto Geográfico Nacional* and *Centro Nacional de Información Geográfica*). The data were obtained for the PNOA project, in autumn 2009 in eastern Galicia (provinces of Lugo and Ourense) and in autumn 2010 in western Galicia (provinces of A Coruña and Pontevedra). The data were acquired with a RIEGL LMS-Q680 sensor, installed in a fixed wing aerial platform, operated at 1064 nm, a pulse repetition rate of 70 kHz, a scan frequency of 46 Hz, a maximum scan angle of ±30°, a maximum beam divergence of 0.5 mrad, an average flying height of 1300 m above sea level, and a minimum swath overlap of 15%. Square ALS blocks of 2 km side and covering the whole region of Galicia were obtained from the CNIG computer server (data available at http://centrodedescargas.cnig.es/CentroDescargas/buscadorCatalogo.do?codFamilia=LIDAR; last accessed on 5-27-2016). A maximum of 4 returns per pulse were registered, with a theoretical laser pulse density required for the PNOA project of 0.5 first returns m^-2^.

#### Extraction of ALS metrics

ALS metrics are descriptive structural statistics calculated from the normalized height of the ALS data cloud (NHD) and processing of raw ALS data is necessary to obtain these metrics. We clipped the ALS cloud within the limit of buffer areas of radius 30 m (25 m of NFI plot plus a buffer of 5 m) and then each cloud was saved as an independent ALS file. We buffered all the selected NFI-4 plots by 5 m to avoid errors (especially near of the plot boundaries) in the subsequent height normalization processes. After that, the outliers were removed considering a window size of 100 m and a maximum and minimum ellipsoidal height bound of ± 5.0*Std. dev. A filtering algorithm (adapted from [33]), based on linear prediction [34], was used to extract ground returns from the ALS point cloud. All returns were used in this process, the cell size was set to 2 m (based on available ALS data density) and the other parameters (a:1.0; b:4.0; g:-2.5; w:2.5; iterations:5) were obtained from Barreiro-Fernández et al. [35], as these performed well with a variety of land cover, forest types and slopes. Then, a 2 m cell size DEM grid was generated by estimating the elevation of each grid cell from the average elevation of all points within the cell; if the cell does not contain any points, it is filled by interpolation from the neighbouring cells. In the following step, the normalized ALS point cloud was obtained by subtraction of the ellipsoidal height of the DEM from the ellipsoidal height of each ALS return; returns below a normalized height of 0 m were excluded. The normalized ALS point cloud within the limits of each field plot (which were previously stored as polygons in shapefiles) were clipped, and an independent file for each plot of 25 m radius was created. Finally, height and canopy cover metrics of the clipped and normalized point clouds were estimated using all returns. The minimum height threshold (*MHT*), which is commonly specified as the lower boundary for calculating height metrics (central tendency, dispersion, shape and percentile statistics), was established at 1.5 m. The height break threshold (*HBT*), which is the limit for separating the point cloud data into two sets to separate canopy returns from the under canopy returns, in order to estimate canopy cover metrics, was established as 4 m (based on field observation). A new filter was applied to remove any remaining outliers; only returns with normalized heights between 0–60 m were included in the analysis, after considering the highest tree apexes observed in the field. In total, 38 metrics widely used to estimate variables related to canopy cover and height distribution [23,36] were extracted from ALS pulses and used as predictors in the statistical analysis. The ALS metrics and the corresponding descriptions for height distribution and canopy cover are summarised in Table 1. Note that all variables were computed from all ALS returns in the database, i.e. all returns per laser pulse.

**Table 1:** Potential explanatory variables related to height distribution and canopy cover.

Variables related to height distribution (m)	Description
*h*_*max*_	maximum
*h*_*mean*_	mean
*h*_*mode*_	mode
*h*_*median*_	median
*h*_*SD*_	standard deviation
*h*_*CV*_	coefficient of variation
*h*_*skw*_	skewness
*h*_*kurt*_	kurtosis
*h*_*ID*_	interquartile distance
*h*_*AAD*_	average absolute deviation
*h*_*MADmedian*_	median of the absolute deviations from the overall median
*h*_*MADmode*_	mode of the absolute deviations from the overall mode
*h*_*L*1_, *h*_*L*2_,*…*, *h*_*L*4_	L-moments
*h*_*Lskw*_	L-moment of skewness
*h*_*Lkur*_	L-moment of kurtosis
*h*_05_, *h*_10_, *h*_20_,…, *h*_90_, *h*_95_, *h*_99_	percentiles
*h*_25_ and *h*_75_	first and third quartiles
Variables related to canopy cover (%) Variables related with height (i)	Description
*PFR*_*Ahmean*_	ratio of the number of the first laser returns above *h*_*mean*_ to the number of first laser returns for each plot
*PFR*_*Ahmode*_	ratio of the number of the first laser returns above *h*_*mode*_ to the number of first returns for each plot
*PAR*_*Ahmean*_	ratio of the number of the all laser returns above *h*_*mean*_ to the number of all laser returns for each plot
*PAR*_*Ahmode*_	ratio of the number of the all laser returns above *h*_*mode*_ to the number of all laser returns for each plot
*PFR*_*A4*_	ratio of the number of the first laser returns above 4 m height to the total number of first laser returns for each plot
*PAR*_*A4*_	ratio of the number of the all laser returns above 4 m height to the total number of first laser returns for each plot

### Quality control and plot selection

The plot positioning procedures used in the NFI-4 were applied by using standard GPS equipment; the plot coordinates therefore had an expected average accuracy of approximately 3–5 m. Gobakken and Næsset [37] observed that the use of larger plot sizes (300–400 m^2^) compensates for errors in positioning sample plots for estimating biophysical properties derived from ALS data. The size of the sample plot used in the NFI is around 1964 m^2^; for positioning errors of 5 and 10 m (much larger than the assumed error), the overlapping areas between a plot in true position and a plot located in an altered position were 84.3 and 74.7%, respectively. Moreover, the precision of the ALS estimates tends to be less sensitive to positioning errors in even-aged plots (as in the present study) and dense forests [37]; therefore, only plots with a crown projection area greater than 90% were selected. The final number of sample plots was 646 (512 *P*. *pinaster* and 134 *P*. *radiata* plots). In Galicia, the NFI-4 measurements were made between 4 and 11 months before ALS measurements. Some of the plots may have been thinned or cropped during this interval, and the ALS metrics would therefore not reflect the stand structure of the plots on the measurement date. In order to prevent errors in the subsequent modelling process, we tried to remove all plots that had undergone silvicultural treatments during the interval between measurements. Visiting all NFI inventory plots is very expensive and time-consuming and was deemed unfeasible. Visualization of ALS data was also ruled out as a means of plot selection because it does not guarantee detection of all disturbed plots during this interval. Finally, the following two (rejection) criteria were used in sample plots selection: 1) all sample plots with a percentage of first returns above 2 m less than 80% were rejected, and 2) plots for which the field-measured dominant height (*H*) and ALS-derived 99th height percentile (*h*_99_) differed by more than 3 times the root mean square error (RMSE) of a pre-existing model for the species and study area were rejected. This model was fitted from a network of 25 permanent plots installed in pine stands through Galicia and related the field-measured *H* to the ALS metric *h*_99_ (for more details, see [23]).

$$\hat{H}=1.06040\cdot h_{99}\;R^{2}=0.9943;\;RMSE=1.826\text{ m}$$ (1)

Taking the second criterion into account, plots with absolute differences between field-measured *H* and ALS-derived *h*_99_ larger than 5.48 m were disregarded for further analysis. Under these decision-making criteria, the number of plots selected was reduced from 646 to 554 (436 for *P*. *pinaster* and 118 for *P*. *radiata*). The mean, maximum, minimum values and standard deviation for the main tree and stand variables for both species are shown in Table 2.

**Table 2:** Descriptive statistics for the main tree and stand variables corresponding to the sample plots used in this study. Std. Dev., standard deviation; *d*, tree diameter; *h*, total tree height; *cl*, crown length (defined as the distance from the crown base to the tree top); *N*, stand density; *dg*, quadratic mean diameter; *G*, stand basal area and *H*, dominant height (estimated as the mean height of the 100 thickest trees per ha).

	Statistics	*d* (cm)	*h* (m)	*cl* (m)	*N* (stems ha^-1^)	*dg* (cm)	*G* (m^2^ha^-1^)	*H* (m)
*Pinus pinaster* (n = 436)	Minimum	7.50	1.50	0.10	159	4.33	3.19	6.10
	Maximum	92.00	45.80	23.70	4615	53.47	83.83	28.75
	Mean	27.87	15.90	6.28	911	19.69	21.88	15.09
	Std. Dev.	12.70	5.94	2.91	735.02	8.30	14.59	5.66
*Pinus radiata* (n = 118)	Minimum	7.50	1.30	0.60	213	4.57	6.52	7.60
	Maximum	107.00	39.30	23.30	3191	84.72	78.28	30.37
	Mean	26.58	17.35	8.13	784	21.45	22.55	16.98
	Std. Dev.	11.28	6.20	3.86	532.86	9.86	13.40	5.70

### Vertical distribution of available canopy fuel estimated from field data

The vertical canopy fuel profiles were constructed by sectioning all trees of each plot in 0.3-m horizontal layers from the ground to the apex of the tallest tree, according to Sando and Wick [5]. The values of available fuel load for these layers were predicted for each selected sample plot by combining estimates from individual-tree crown profile models and from a system of biomass models.

The crown architecture of each tree was estimated using the crown profile systems developed for radiata pine and maritime pine by [38] and [39], respectively. These systems include two models: one for the upper crown of the tree (above the maximum crown radius) and another for the lower crown (below the maximum crown radius). The input variables for these models are *d*, *h*, *G*, *H*, *t* and *S* (*t* is the stand age (years) and *S* is the site index, estimated as the dominant height of the stand at a reference age of 20 years). The outputs of the system are the individual crown base height (*cbh*), the crown length (*cl*), and the crown radius (*cr*) at different points along the crown.

In the present study, we first estimated the upper and lower crown radii for 0.3-m layers above ground for each tree of the sample plots. We then predicted the volume of the *i*th crown section (*crV*_i_, m^3^) using the Smalian’s approximation for the volume of a frustum of a second degree paraboloid, as follows:

$$crV_{i}=\frac{\pi \cdot \left(cr_{up}{}_{i}^{2}+cr_{low}{}_{i}^{2}\right)}{2}\cdot 0.3$$ (2)

where *cr*_*upi*_ is the upper and *cr*_*lowi*_ the lower crown radius (m) of the *i*th layer (0.3 m thickness).

The available fuel load of each crown section was estimated by assuming that the fine biomass is distributed vertically according to the vertical distribution of the crown volume.

The compatible systems of tree biomass models developed for maritime pine and radiata pine in Galicia [40] were used to estimate the available fuel for each tree as the sum of needles and twigs (0.6-cm maximum butt diameter). These systems require that measurements of *d* and *h* are available for each tree.

The vertical profile of available *CFL* for each plot was estimated by summing the available fuel weight in 0.3-m vertical layers across all trees and dividing by the plot area. The minimum canopy base height (*mCBH*) was defined as the height at the lower limit of the first 0.3-m layer with a value of *CFL* greater than zero.

A two-parameter Weibull probability density function was used to characterize the vertical profiles of available *CFL*. The Weibull function has previously been used in ALS-based studies for estimating diameter distributions (e.g. [24–27]):

$$\begin{array}{l} CFL_{i}=CFL\left(\frac{a_{2}}{a_{1}}\right){\left(\frac{CL_{i}-mCBH}{a_{1}}\right)}^{a_{2}-1}e^{{\left(\frac{CL_{i}-mCBH}{a_{1}}\right)}^{a_{2}}}\\ CFLrel_{i}=\frac{CFL_{i}}{CFL} \end{array}$$ (3)

where *CFL*_*i*_ is the canopy fuel load for the layer *i* (kg m^-2^); *CFLrel*_i_ is the ratio between the available *CFL* of the layer *i* and the total *CFL*; *CFL* is the total canopy fuel load (kg m^-2^); *CL*_*i*_ is the canopy length for the layer *i* (m), defined as the distance from *mCBH* to the upper limit of the 0.3-m layer (m); and *a*_1_ and *a*_2_ are the scale and shape parameters of the Weibull function, respectively. The Weibull parameters *a*_1_ and *a*_2_ were estimated using the first (*m*_1_) and the second (*m*_2_) moments of the relative *CFL* distribution (*CFLrel*) [41]:

$$m_{2}=\frac{{\left(m_{1}-mCBH\right)}^{2}}{\Gamma^{2}\left[1+\frac{1}{a_{2}}\right]}\cdot \left(\Gamma \left[1+\frac{2}{a_{2}}\right]-\Gamma^{2}\left[1+\frac{1}{a_{2}}\right]\right)$$ (4)

$$a_{1}=\frac{m_{1}-mCBH}{\Gamma \left[1+\frac{1}{a_{2}}\right]}$$ (5)

where $m_{1}=\sum_{i=1}^{num.\;layers}CL_{i}\cdot CFLrel_{i}$; $m_{2}=\sum_{i=1}^{num.\;layers}CL_{i}\cdot CFLrel_{i}^{2}-{\left(\sum_{i=1}^{num.\;layers}CL_{i}\cdot CFLrel_{i}\right)}^{2}$; Γ is the Gamma function. Eq 4 can be resolved iteratively for parameter *a*_2_, and parameter *a*_1_ is then predicted using Eq 5.

### Modelling the vertical distribution of available canopy fuel

The vertical profiles of available canopy fuel at stand level can be estimated from ALS variables by fitting four models for each species to estimate (i) the total canopy fuel load (*CFL*), (ii) the minimum canopy base height (*mCBH*), (iii) the scale parameter of the Weibull function (*a*_1_), and (iv) the shape parameter of the Weibull function (*a*_2_). The Weibull parameters (*a*_1_ and *a*_2_) can be directly related to ALS variables or indirectly estimated by relating the first and the second moments of the relative *CFL* distribution to the ALS metrics and then using Eqs 4 and 5 to estimate the values.

Linear (Eq 6) and (multiplicative) power (Eq 7) models were used to fit the four relationships for each species:

$${\hat{y}}_{i}=\beta_{0}+\beta_{1}x_{1i}+\beta_{2}x_{2i}+\cdots +\beta_{k}x_{ki}+\varepsilon_{i}$$ (6)

$${\hat{y}}_{i}=\beta_{0}x_{1i}^{\beta_{1}}x_{2i}^{\beta_{2}}\cdots x_{ki}^{\beta_{k}}\varepsilon_{i}$$ (7)

where *β*_*j*_ (*j* = 1,…,*k*) are the parameters to be estimated; *x*_*ji*_ is the observation of the *j*th predictor on the *i*th sample plot; and *ε*_*i*_ is the error.

Model fitting was carried out in two steps. In the first step, the set of predictors (ALS metrics or stand variables) for each dependent variable in models (6) and (7) was selected by using the stepwise variable selection method, in combination with an understanding of the process modelled (biological sense of the variables and the signs of the parameter estimates). In order to avoid multicollinearity and over-fitted models, predictors with a condition number above 30 were disregarded [42]. In addition, the absence of heteroscedasticity was tested using the White-test [43] (weighted regression would be applied if heteroscedasticity were detected). The power model (Eq 7) was initially linearized to use the stepwise variable selection method and, once the set of predictors was selected, the original model was fitted using nonlinear regression with the parameter estimates of the linear model as initial values of the iterative procedure.

Fitted models (6) and (7) were subsequently compared for each dependent variable on the basis of graphical analysis of observed against predicted values of the dependent variable and the studentized residuals against the predicted values of the dependent variable. The goodness-of-fit statistics RMSE and the model efficiency (ME) were also calculated:

$$\text{RMSE}=\sqrt{\frac{{\sum_{i=1}^{n}\left(y_{i}-{\hat{y}}_{i}\right)}^{2}}{n-p}}$$ (8)

$$\text{ME}=\text{ 1-}\frac{(n-1)\sum_{i=1}^{n}{\text{(}y_{i}-{\hat{y}}_{i})}^{2}}{(n-p)\sum_{i=1}^{n}{\text{(}y_{i}-\bar{y})}^{2}}$$ (9)

where *y*_*i*_, ${\hat{y}}_{i}$ and $\bar{y}$ are the observed, predicted and mean values of the dependent variable, respectively, *n* is the total number of observations, and *p* is the number of model parameters.

In a second step, the four selected models for each species were fitted simultaneously by use of the full information maximum likelihood (FIML) method implemented in the SAS/ETS^®^ Model Procedure [44]. Simultaneous fitting must be carried out because the values of the four dependent variables were estimated for each sample plot from the same profile of vertical available *CFL*, and the residuals are therefore expected to be correlated. The FIML method takes into account the cross-model correlations, thus improving the efficiency of the estimation. In the first step, the cross-model error covariance matrix required for initiating the iterative procedure was estimated using the ordinary least squares (OLS) method.

In order to assess the error involved when the proposed approach for estimating the vertical available *CFL* profiles is used for prediction, the RMSE and the ME (Eqs 8 and 9) were calculated by comparing the vertical profiles estimated from field data and the vertical profiles estimated from ALS metrics for each sample plot. Standard goodness-of-fit tests often used to evaluate the performance of distribution functions (e.g. Kolmogorov-Smirnov, Cramér-von Mises and Andersen-Darling tests) were not used because (i) Weibull parameters were estimated from the data using a relatively complicated approach, and the critical values of the tests are therefore not valid [45,46], and (ii) some tests may not provide much useful information about the performance of the model as they are not designed to answer questions about the error associated with estimates and predictions obtained from the model. Rejecting the null hypothesis of these tests signifies that a model is not a perfect representation of the observed distribution; however, although not perfect this may be the most accurate available model [46].

## Results

### Vertical distribution of *CFL*

**Fig 3:**
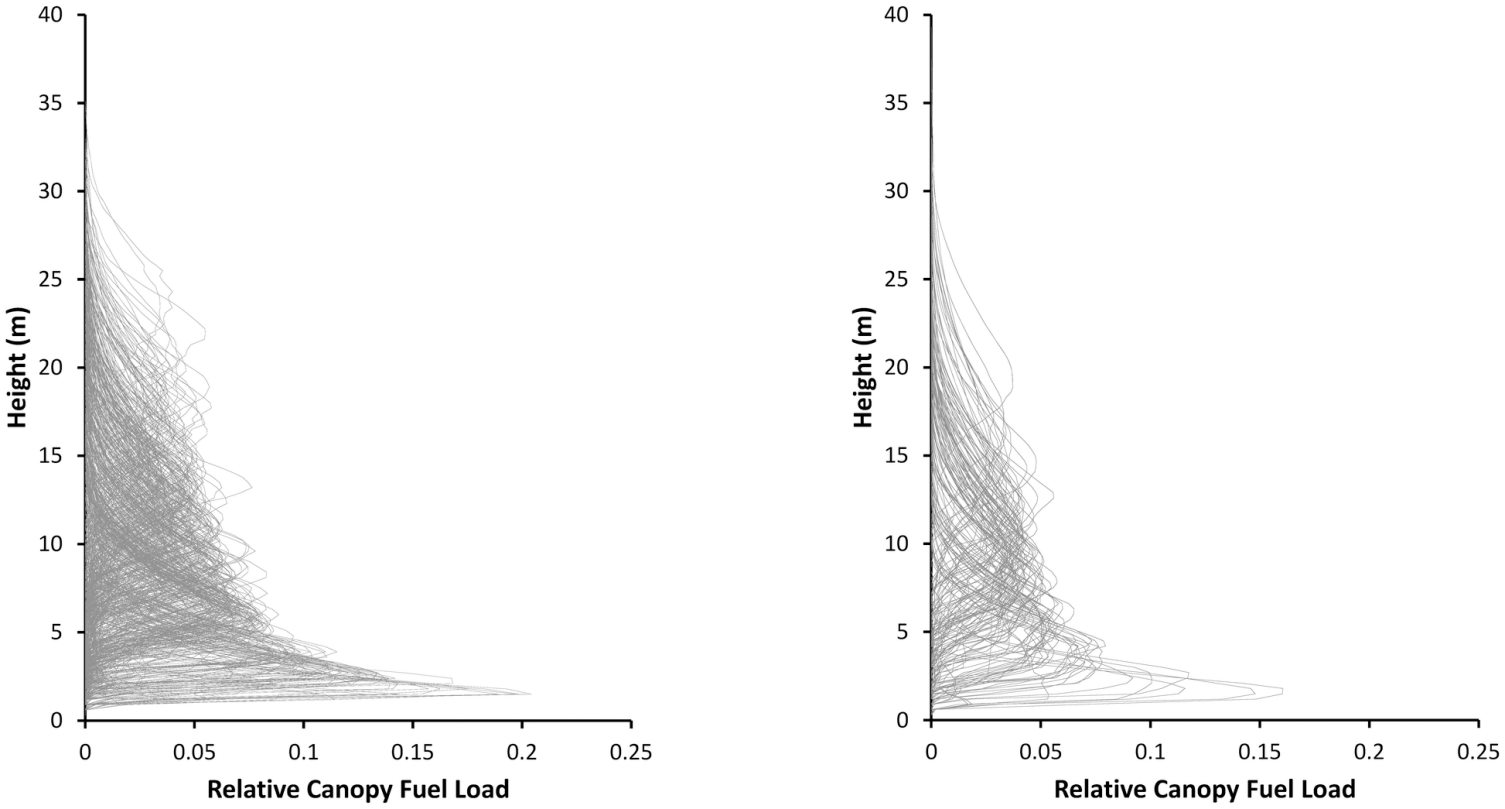
Vertical profiles of relative canopy fuel load (*CFLrel*) in each sample plot of *P*. *pinaster* (left) and *P*. *radiata* (right) estimated from field data.

The vertical profiles of *CFLrel* in each sample plot are shown in Fig 3. Eqs (4) and (5) were used to estimate the scale and shape parameters of the Weibull function (*a*_1_ and *a*_2_, respectively) for each sample plot.

**Fig 4:**
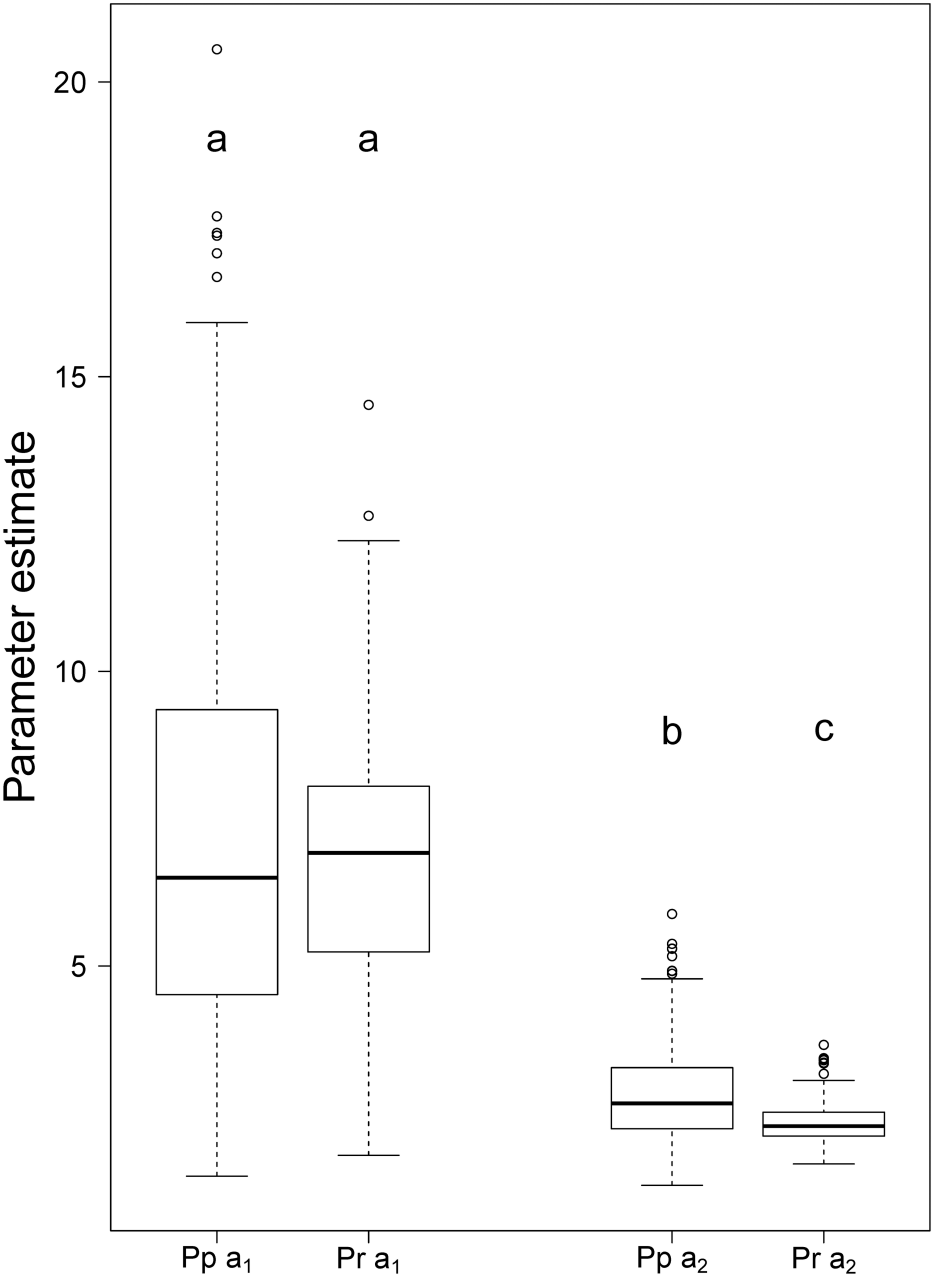
Box plot of the estimated values of the scale and shape parameters of the Weibull function (*â*_1_ and *â*_2_, respectively) for *P*. *pinaster* (Pp) and *P*. *radiata* (Pr) sample plots. Different letters indicate significant differences between mean values (*α* = 0.05).

The values of *â*_1_ ranged from 1.44 to 20.56 with a mean value of 7.22 (Std. Dev = 3.63) for maritime pine, and from 1.79 to 14.52, with a mean value of 6.81 (Std. Dev = 2.32), for radiata pine. The values of *â*_2_ ranged from 1.28 to 5.88, with a mean value of 2.81 (Std. Dev = 0.77) for maritime pine, and from 1.64 to 3.67, with a mean value of 2.37 (Std. dev = 0.39) for radiata pine. We used Tukey’s adjusted pairwise comparisons to study the influence of pine species on both parameters and found that the mean values of *â*_1_ and *â*_2_ were greater for maritime pine (Fig 4), although only the differences in parameter *â*_2_ were significant (*F*-value 36.33; *p* <0.0001).

The estimated Weibull parameters and the estimated *CFL* and *mCBH* from field data were used to obtain estimated vertical distributions of *CFL*. These distributions explained more than 99% of the variation in the *CFL* vertical distributions estimated from field data for both species with a RMSE value of 0.0159 (RMSE% relative to the mean *CFL* value = 2.78%) for maritime pine and 0.0150 (RMSE% relative to the mean *CFL* value = 2.20%) for radiata pine.

### System of models based on stand variables

The first system of fitted models for estimating the vertical distribution of canopy fuel loads uses stand variables as predictors. Number of trees per hectare, stand basal area, quadratic mean diameter and dominant height were used as predictors. The system of models for each species, obtained by simultaneous fitting, is shown in Table 3.

**Table 3:** Parameter estimates and goodness-of-fit statistics for the systems of models obtained for each species by using stand variables as predictors. ME is the model efficiency (Eq 9) and RMSE is the root mean square error (Eq 8). All parameter estimates were significant at *p* < 0.05.

Species	Dependent variable	Model	${\hat{b}}_{0}$	${\hat{b}}_{1}$	${\hat{b}}_{2}$	ME	RMSE
*P*. *pinaster*	Canopy Fuel Load (*CFL*, kg m^-2^)	$C\hat{F}L={\hat{b}}_{1}G$	---	0.0265	---	0.9668	0.0747
	Minimum Canopy Base Height (*mCBH*, m)	$mC\hat{B}H={\hat{b}}_{0}H^{{\hat{b}}_{1}}dg^{{\hat{b}}_{2}}$	0.0107	0.7615	1.2144	0.6444	1.4748
	First Moment (*m*_1_)	${\hat{m}}_{1}={\hat{b}}_{0}H^{{\hat{b}}_{1}}dg^{{\hat{b}}_{2}}$	0.3752	1.0792	0.1153	0.9608	0.8775
	Second Moment (*m*_2_)	${\hat{m}}_{2}={\hat{b}}_{0}H^{{\hat{b}}_{1}}dg^{{\hat{b}}_{2}}$	0.3946	1.9370	-0.8420	0.6448	3.3604
*P*. *radiata*	Canopy Fuel Load (*CFL*, kg m^-2^)	$C\hat{F}L={\hat{b}}_{1}G$	---	0.0299	---	0.9847	0.0488
	Minimum Canopy Base Height (*mCBH*, m)	$mC\hat{B}H={\hat{b}}_{0}H^{{\hat{b}}_{1}}G^{{\hat{b}}_{2}}$	0.0306	1.3747	0.2493	0.7045	1.3003
	First Moment (*m*_1_)	${\hat{m}}_{1}={\hat{b}}_{0}H^{{\hat{b}}_{1}}G^{{\hat{b}}_{2}}$	0.3092	1.1326	0.0649	0.9637	0.7477
	Second Moment (*m*_2_)	${\hat{m}}_{2}={\hat{b}}_{0}H^{{\hat{b}}_{1}}G^{{\hat{b}}_{2}}$	0.1055	1.7609	-0.2286	0.7418	2.4237

Note: *H* = dominant height (m), *G* = stand basal area (m^2^ha^-1^), *dg* = quadratic mean diameter (cm)

According to the values of the goodness-of-fit statistics, linear models were more accurate than powered models for *CFL* in both species, whereas powered models were more accurate for the other three dependent variables. The values of the condition numbers and the results of the White-test indicated the absence of any problems related to multicollinearity or heteroscedasticity.

The observed variation explained by the models of *CFL* and *mCBH* ranged from 64% to 98%, with slightly greater values for radiata pine stands. The models used to estimate the first moment explained more than 96% of the observed variation, whereas the models of the second moment explained 64% and 74% of the observed variation for maritime pine and radiata pine, respectively.

Dominant height was the main predictor in all models in which the dependent variable was directly or indirectly related to canopy height (*mCBH* and the moments of the Weibull distribution); moreover, when other predictor variables were also included in those models, only quadratic mean diameter for maritime pine and stand basal area for radiata pine significantly improved the results obtained with dominant height. The only predictor in the *CFL* models was basal area for both species.

The system of four models was used to estimate the vertical distributions of *CFL* of each sample plot. The profiles obtained explained 84% and 86% of the variation for maritime pine and radiata pine respectively, with a RMSE value of 0.1441 for maritime pine (RMSE% relative to the mean *CFL* value = 25.29%) and of 0.1518 for radiata pine (RMSE% relative to the mean *CFL* value = 22.32%).

**Fig 5:**
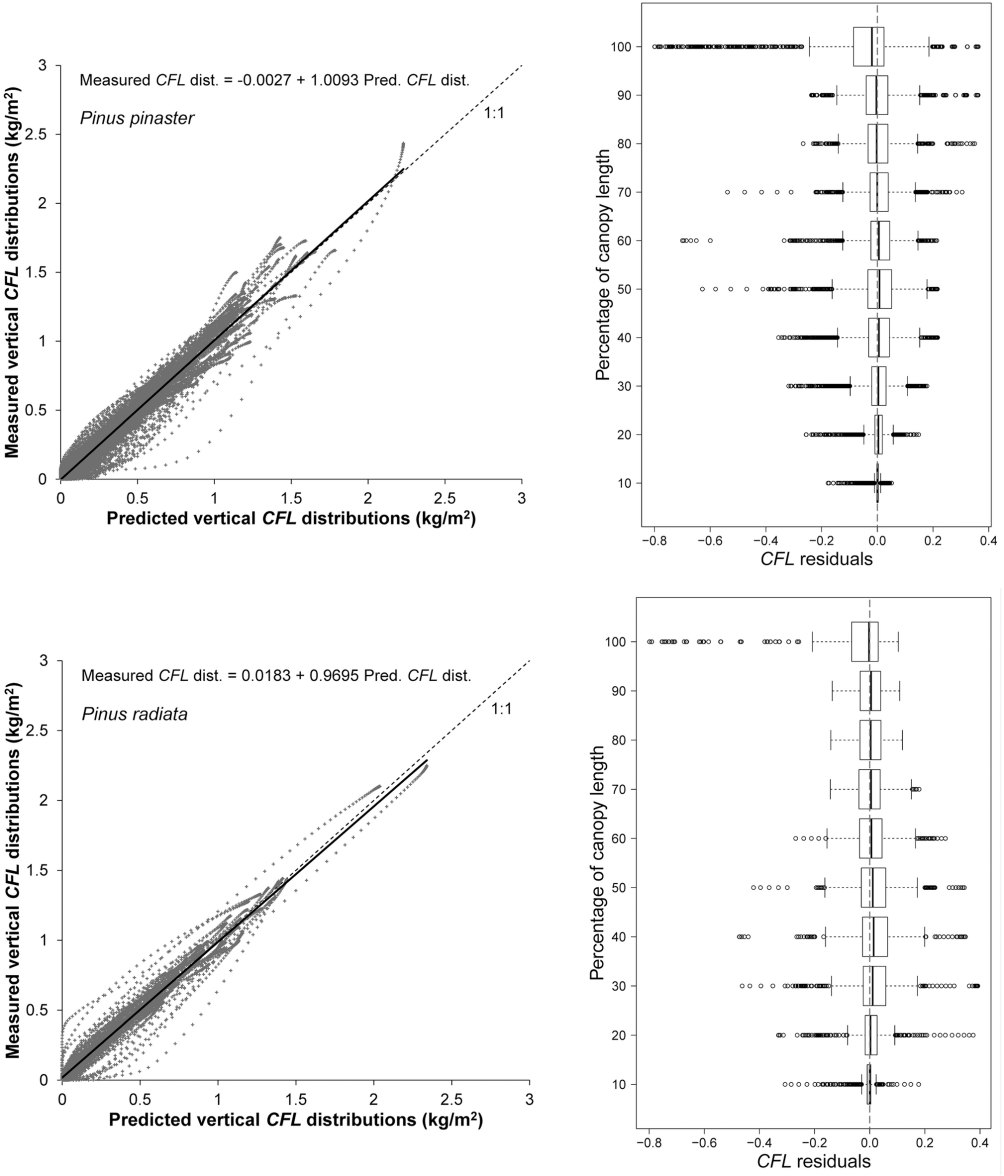
Scatter diagrams of vertical *CFL* distributions estimated from field data against predicted vertical *CFL* distributions obtained using stand variables as predictors. The solid line represents the linear model fitted, and the broken line is the 1:1 line (left, first column). Box plot of *CFL* residuals (using stand variables as predictors) against canopy length classes (right, second column). Graphs for *P*. *pinaster* are in the first row and for *P*. *radiata* in the second row.

Graphs of *CFL* distributions estimated from field data against predicted *CFL* distributions using the system based on stand variables and box plots of residuals against percentage of canopy length obtained with the system based on stand variables are shown in Fig 5. The scatter diagrams indicated that both distributions were strongly correlated and did not tend to be either over- or underestimated. Moreover, examination of the box plots showed than the residuals were homogeneously distributed along the canopy length and the greatest residuals were located in the upper 10% of the canopy.

### System of models based on ALS variables

Finally, a system of four models was fitted for each species to predict the vertical distribution of *CFL* using ALS metrics as predictors. The combination of models (linear or power) and predictors obtained in the simultaneous fitting are shown in Table 4.

**Table 4:** Parameter estimates and goodness-of-fit statistics for the systems of models obtained for each species using ALS metrics as predictors. ME is the model efficiency (Eq 9) and RMSE is the root mean square error (Eq 8). All parameter estimates were significant at *p* < 0.05.

Species	Dependent variable	Model	${\hat{b}}_{0}$	${\hat{b}}_{1}$	${\hat{b}}_{2}$	*ME*	*RMSE*
*P*. *pinaster*	Canopy Fuel Load (*CFL*, kg m^-2^)	$C\hat{F}L={\hat{b}}_{0}h_{99}^{{\hat{b}}_{1}}PFR_{Ahmean}^{{\hat{b}}_{2}}$	0.0443	0.9057	0.0631	0.4132	0.3145
	Minimum Canopy Base Height (*mCBH*, m)	$mC\hat{B}H={\hat{b}}_{0}h_{99}^{{\hat{b}}_{1}}$	0.4144	0.7998	---	0.4863	2.1030
	First Moment (*m*_1_)	${\hat{m}}_{1}={\hat{b}}_{0}h_{99}^{{\hat{b}}_{1}}$	0.9401	0.8798	---	0.7616	2.1616
	Second Moment (*m*_2_)	${\hat{m}}_{2}={\hat{b}}_{0}h_{99}^{{\hat{b}}_{1}}$	0.1548	1.3852	---	0.4747	4.0823
*P*. *radiata*	Canopy Fuel Load (*CFL*, kg m^-2^)	$C\hat{F}L={\hat{b}}_{1}h_{99}+{\hat{b}}_{2}PFR_{Ahmean}$	---	0.0367	0.0058	0.4583	0.2910
	Minimum Canopy Base Height (*mCBH*, m)	$mC\hat{B}H={\hat{b}}_{1}h_{99}$	---	0.2222	---	0.5225	1.6388
	First Moment (*m*_1_)	${\hat{m}}_{1}={\hat{b}}_{0}h_{99}^{{\hat{b}}_{1}}$	1.0223	0.8108	---	0.7288	2.0340
	Second Moment (*m*_2_)	${\hat{m}}_{2}={\hat{b}}_{0}h_{99}^{{\hat{b}}_{1}}$	0.2321	1.2650	---	0.5317	3.2499

Note: *h*_99_ = height of the 99^th^ percentile of ALS returns (m), *PFR*_*Ahmean*_ = percentage of first returns above mean.

According to the goodness-of-fit statistics, linear models were more accurate than power models for *CFL* and *mCBH* in radiata pine, whereas the power models were more accurate for the other models. The values of the condition numbers and the results of the White-test did not indicate any problems related to multicollinearity or heteroscedasticity.

The variation explained by the models of *CFL* and *mCBH* ranged from 41% to 52%, and the values were again slightly greater for radiata pine stands. The models for the first moment explained more than 72% and 76% of the observed variation, and the models for the second moment explained more than 47% and 53% of the observed variation, for respectively maritime and radiata pine. The height of the 99^th^ percentile of the ALS data was the only predictor in all the models, except the *CFL* model, in which the percentage of first returns above mean was also included for both species.

This system of four models was also used to estimate the vertical distributions of *CFL* for each sample plot. The profiles obtained explained 52% and 49% of the variation for maritime pine and radiata pine respectively, with a RMSE value of 0.2587 for maritime pine (RMSE% relative to the mean *CFL* value = 45.39%) and 0.3028 for radiata pine (RMSE% relative to the mean *CFL* value = 44.53%).

**Fig 6:**
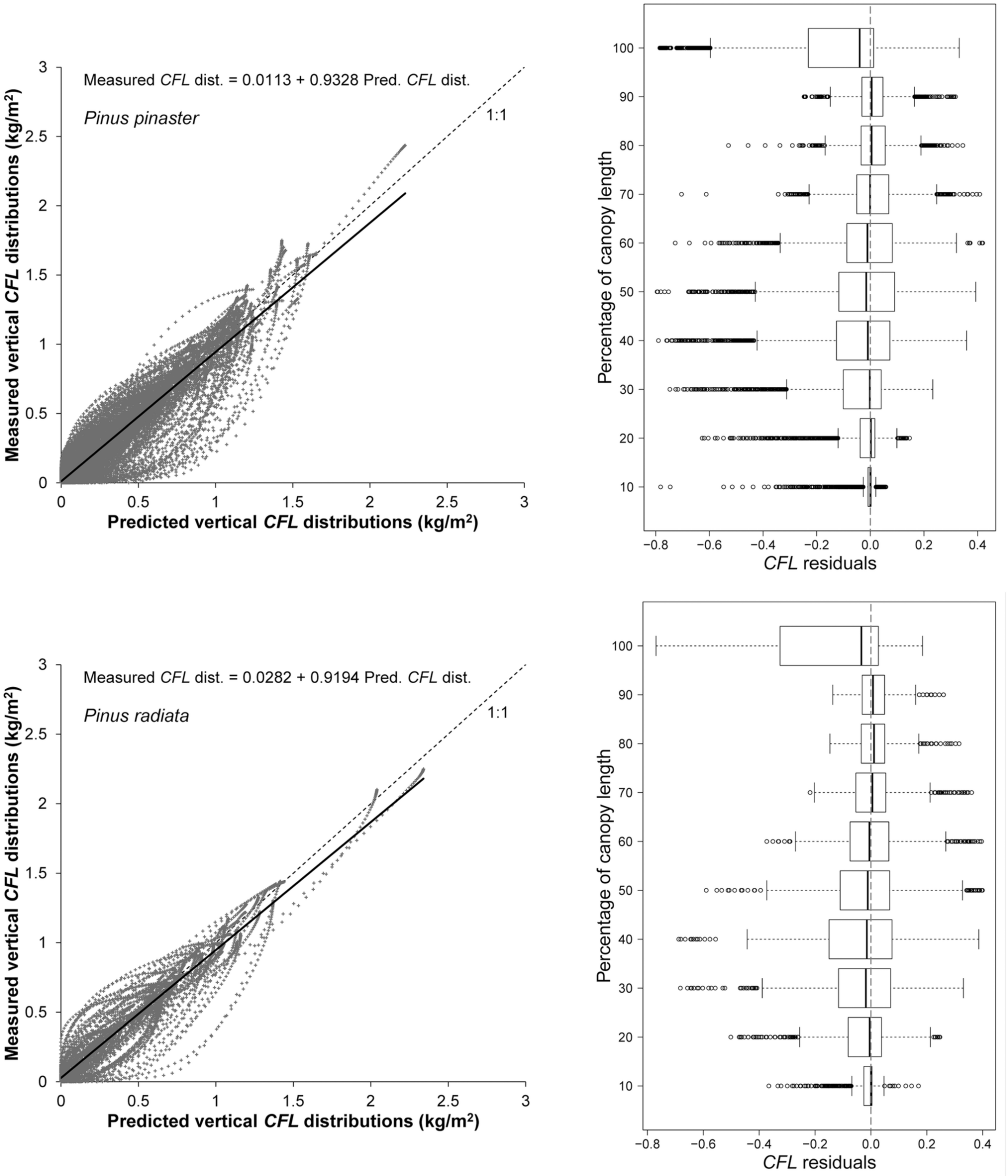
Scatter diagram of vertical *CFL* distributions estimated from field data against vertical *CFL* distributions predicted using ALS metrics. The solid line represents the linear model fitted, and the dashed line is the 1:1 line (left, first column). Box plot of *CFL* residuals (using ALS metrics as predictors) against canopy length classes (right, second column). Graphs for *P*. *pinaster* are in the first row and those for *P*. *radiata* are in the second row.

Graphs of *CFL* distributions estimated from field data against predicted *CFL* distributions using the system based on ALS metrics and box plots of residuals against percentage of canopy length obtained with the system based on ALS variables are shown in Fig 6 for each species. The scatter diagrams indicated a slight tendency for the *CFL* distributions to be overestimated for both species. In the same way as for the system of models based on stand variables, inspection of the box plot indicated that the residuals were homogeneously distributed along the canopy length, and the largest residuals were located in the upper 10% of the canopy.

## Discussion

The two systems developed here provide researchers and forest fuel load managers with useful tools for describing the structural characteristics of the canopy fuel complex in *P*. *pinaster* and *P*. *radiata* stands. Moreover, these canopy fuel characteristics can be estimated from two different sources of data: from ALS metrics or from forest stand variables measured in the field.

The system based on the use of stand variables as predictors fitted the data more accurately than the system based on ALS metrics. There are two possible reasons for these results: the coarse resolution of the ALS data used (0.5 first returns m^-2^) and the inaccuracy in locating the ground plots. Concerning the first possibility, according to White et al. [36], a minimum of 1 pulse m^-2^ (> 4 pulses m^-2^ for dense forests on complex terrain) is recommended to produce an operational ALS-based enhanced forest inventory. This hypothesis is consistent with the results (*CFL*: *ME* = 0.97, *RMSE* = 0.0363 kg m^-2^; *mCBH*: *ME* = 0.94, *RMSE* = 0.329 m) obtained by Hevia et al. [47] in maritime pine stands in Asturias (a region bordering Galicia) with an ALS sampling density ranging from 8 to 16 first returns m^-2^.

Concerning the second possibility, White el al. [36] pointed out the need for accurate georeferencing of ground plots to maximize the predictive power of the models developed. In the present study, the 99^th^ height percentile (*h*_99_) of the points cloud was the only ALS predictor in all models except *CFL*, in which the percentage of first returns above mean (*PFR*_*Ahmean*_) was also included as a predictor. According to the results obtained by Gobakken and Næesset [37], both *h*_99_ and *PFR*_*Ahmean*_ are two of the ALS metrics least affected by plot georeferencing errors. These authors estimated a mean error smaller than 0.5 m and less than 2% for *h*_99_ and *PFR*_*Ahmean*_, respectively working with plots of 300–400 m^2^ and a georeferencing error of the center of the plot of 10 m. As an example, the system of four models based on ALS metrics developed in the present study was used to estimate the vertical distributions of *CFL* of each sample plot considering the above mentioned errors for each predictor, yielding reductions of 1.2% and 0.9% in the *RMSE* for maritime pine and radiata pine, respectively. Moreover, taking into account the larger surface area of the sample plots used in the present study (1964 m^2^) and assuming that the plot georeferencing errors are smaller than 10 m, the effect of this source of error on the accuracy of the estimates can be considered negligible.

One remarkable characteristic of the systems of models developed is that they are compatible with the two main approaches used to define and estimate canopy fuel variables related to crown fire initiation and spread (the simplest one, which assumes that available canopy fuel is homogeneously distributed throughout the aerial layer, and the more complex and realistic ones, which assume that the crown shape defines the available fuel distribution along the crown). Moreover, modelling the available canopy fuel load vertical profile (necessary for the latter approach) enables *CBH* to be estimated for any of the canopy bulk density thresholds defined to date or for new values that may be defined by crown fire behavior research in the future.

Maps of the spatial distribution of the canopy variables related to crown fire risk (*CFL*, *CBD* and *CBH*) can be generated by applying the system based on ALS variables using the wall-to-wall ALS metrics. Maritime and radiata pine forest should first be identified by using the strata of the Spanish forest map (data available at http://www.mapama.gob.es/es/biodiversidad/servicios/banco-datos-naturaleza/informacion-disponible/mfe50.aspx). Estimates according to the more realistic fuel vertical distribution approach require the use of the whole system of four models. When the fire simulator used requires estimates based on the simple approach, mean stand height ($\bar{h}$) should also be predicted. In this case, the models previously developed for the species in the study area to estimate $\bar{h}$ from ALS metrics should be used [23,47,48].

The proposed models could be used to evaluate the effects of different forest management alternatives for reducing crown fire hazard. Thinning and pruning treatments should be applied in order to increase *CBH* and reduce *CBD* below 0.1 kg m^-3^, the accepted threshold for crown fire propagation [1], thus yielding stand structures that are more resistant to the initiation and spread of crown fire [7]. In addition to information about vertical canopy fuel distribution, fuel management decisions must also be based on values of other essential variables such as surface fuel load and moisture content, foliar moisture content and wind reduction by the canopy and topography [2].

## Conclusions

Data from the Spanish National Forest Inventory and low density ALS data were used to predict the vertical distribution of canopy fuel load in *P*. *radiata* and *P*. *pinaster* stands in Galicia (north-west Spain). Two different systems of models, one using stand variables and the other using ALS metrics as predictors, have been proposed. Models with stand variables as predictors performed better than those with ALS metrics, probably due to the coarse resolution of ALS data used. The main advantage of the system of models based on ALS metrics is that these inputs are available for all population elements, and could be used to predict the vertical distribution of *CFL* over the entire area of the ALS data coverage. Consequently, maps representing spatially explicit data layers of canopy fuel variables (*CFL*, *CBH*, *CBD*) can be obtained and subsequently used as inputs for fire behaviour simulators.

Recent advances in modelling crown fire behavior require accurate estimates of the vertical distribution of canopy fuels, and the models developed can therefore be used to good advantage. Nevertheless, other important areas of forest research such as carbon accounting, selective biomass estimation from pruning and thinning treatments, ecological modelling of the light regime within the crown and physiological modelling of canopy photosynthesis would also benefit greatly from better knowledge of vertical distribution of tree crown biomass [49].
